# *N*-(4-Chloro­phen­yl)-*N*′-(3-methyl­phen­yl)succinamide monohydrate

**DOI:** 10.1107/S1600536811032685

**Published:** 2011-08-27

**Authors:** B. S. Saraswathi, Sabine Foro, B. Thimme Gowda

**Affiliations:** a Department of Chemistry, Mangalore University, Mangalagangotri 574199, Mangalore, India; b Institute of Materials Science, Darmstadt University of Technology, Petersenstrasse 23, D-64287, Darmstadt, Germany

## Abstract

In the title hydrate, C_17_H_17_ClN_2_O_2_·H_2_O, the dihedral angles formed by the aromatic rings of the chloro­benzene and methyl­benzene groups with the mean planes of their attached NH—C(O)—CH_2_ fragments are 16.6 (2) and 22.8 (2)°, respectively. In the crystal, O—H⋯O and N—H⋯O hydrogen bonds link the components into a two-dimensional network parallel to the *ab* plane.

## Related literature

For studies on the effects of substituents on the structures and other aspects of *N*-(ar­yl)-amides, see: Arjunan *et al.* (2004 ▶); Gowda *et al.* (2000 ▶); Saraswathi *et al.* (2011 ▶), on *N*-(ar­yl)-methane­sulfonamides, see: Gowda *et al.* (2007 ▶) and on *N*-chloro-aryl­sulfonamides, see: Gowda & Kumar (2003 ▶).
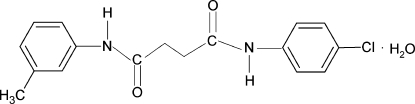


## Experimental

### 

#### Crystal data

C_17_H_17_ClN_2_O_2_·H_2_O
*M* *_r_* = 334.79
Monoclinic, 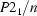

*a* = 12.210 (1) Å
*b* = 4.9111 (5) Å
*c* = 27.078 (3) Å
β = 93.104 (9)°
*V* = 1621.3 (3) Å^3^
*Z* = 4
Mo *K*α radiation
μ = 0.25 mm^−1^
*T* = 293 K
0.36 × 0.28 × 0.08 mm

#### Data collection

Oxford Diffraction Xcalibur diffractometer with Sapphire CCD detector
Absorption correction: multi-scan (*CrysAlis RED*; Oxford Diffraction, 2009 ▶) *T*_min_ = 0.915, *T*_max_ = 0.980
5592 measured reflections
3297 independent reflections
2047 reflections with *I* > 2σ(*I*)
*R*_int_ = 0.027

#### Refinement

*R*[*F* ^2^ > 2σ(*F* ^2^)] = 0.053
*wR*(*F* ^2^) = 0.127
*S* = 1.02
3297 reflections
221 parameters
2 restraints
H atoms treated by a mixture of independent and constrained refinement
Δρ_max_ = 0.37 e Å^−3^
Δρ_min_ = −0.21 e Å^−3^

### 

Data collection: *CrysAlis CCD* (Oxford Diffraction, 2009 ▶); cell refinement: *CrysAlis RED* (Oxford Diffraction, 2009 ▶); data reduction: *CrysAlis RED*; program(s) used to solve structure: *SHELXS97* (Sheldrick, 2008 ▶); program(s) used to refine structure: *SHELXL97* (Sheldrick, 2008 ▶); molecular graphics: *PLATON* (Spek, 2009 ▶); software used to prepare material for publication: *SHELXL97*.

## Supplementary Material

Crystal structure: contains datablock(s) I, global. DOI: 10.1107/S1600536811032685/ds2132sup1.cif

Structure factors: contains datablock(s) I. DOI: 10.1107/S1600536811032685/ds2132Isup2.hkl

Additional supplementary materials:  crystallographic information; 3D view; checkCIF report

## Figures and Tables

**Table 1:** Hydrogen-bond geometry (Å, °)

*D*—H⋯*A*	*D*—H	H⋯*A*	*D*⋯*A*	*D*—H⋯*A*
N1—H1*N*⋯O3^i^	0.85 (2)	2.14 (2)	2.971 (3)	166 (3)
N2—H2*N*⋯O1^ii^	0.85 (2)	2.10 (2)	2.949 (3)	172 (3)
O3—H31⋯O2^iii^	0.88 (3)	1.86 (3)	2.730 (3)	177 (3)
O3—H32⋯O3^i^	0.83 (3)	1.97 (3)	2.802 (2)	175 (3)

Symmetry codes: (i) 
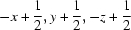
; (ii) 
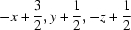
; (iii) 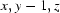.

**Fig. 1.**
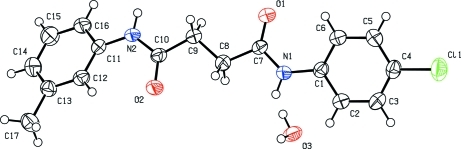
The asymmetric unit of the title compound with displacement ellipsoids drawn at the 50% probability level. The intermolecular O—H···O hydrogen bond involving the water molecule is drawn as a dashed line.

**Fig. 2.**
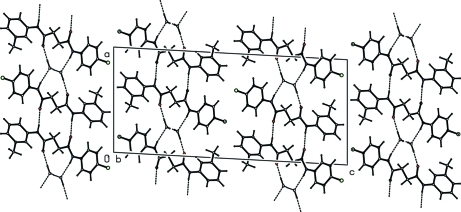
A partial packing diagram of the title compound viewed along the b axis, showing the hydrogen-bonding scheme with dashed lines.

## supplementary crystallographic information

### Comment

The amide and sulfonamide moieties are important constituents of many biologically important compounds. As part of our studies on the effects of substitutions on the structures and other aspects of *N*-(aryl)-amides (Arjunan *et al.*, 2004; Gowda *et al.*, 2000; Saraswathi *et al.*, 2011); *N*-(aryl)-methanesulfonamides (Gowda *et al.*, 2007) and on *N*-chloro-arylsulfonamides (Gowda & Kumar, 2003), in the present work, the structure of *N*-(4-Chlorophenyl),*N*-(3-methylphenyl)-succinamide monohydrate has been determined (Fig.1). In the C—NH—C(O)—C—C—C(O)—NH—C segment of the structure, all the N—H, C=O and C—H bonds in the amide and aliphatic fragments are *anti* to the adjacent bonds, similar to that observed in *N*-(3-chlorophenyl),*N*-(3-methylphenyl)- succinamide (II) (Saraswathi *et al.*, 2011).

Further, conformations of the N—H bond in the amide fragment is *anti* to the *meta*-methyl group in the adjacent benzene ring, similar to that observed in (II). Further, the dihedral angle between the 4-chlorophenyl ring and the adjacent NH—C(O)—CH_2_ segment is 16.6 (2)° and that between the 3-methylphenyl ring and the adjacent NH—C(O)—CH_2_ segment is 22.8 (2)°.

The crystal packing of (I) through N1—H1N···O3, N2—H2N···O1, O3—H31O···O2 and O3—H32O···O3 hydrogen bonding (Table 1) is shown in Fig.2.

### Experimental

Succinic anhydride (0.01 mol) in toluene (25 ml) was treated drop wise with *m*-toluidine (0.01 mol) also in toluene (20 ml) with constant stirring. The resulting mixture was stirred for one hour and set aside for an additional hour at room temperature for completion of the reaction. The mixture was then treated with dilute hydrochloric acid to remove unreacted *m*-toluidine. The resultant solid *N*-(3-methylphenyl)-succinamic acid was filtered under suction and washed thoroughly with water to remove the unreacted succinic anhydride and succinic acid. The compound was recrystallized to constant melting point from ethanol. The purity of the compound was checked and characterized by its infrared and NMR spectra.

The *N*-(3-methylphenyl)-succinamic acid obtained was then treated with phosphorous oxychloride and excess of 4-chloroaniline at room temperature with constant stirring. The resultant mixture was stirred for 4 h, kept aside for additional 6 h for completion of the reaction and poured slowly into crushed ice with constant stirring. It was kept aside for a day. The resultant solid, *N*-(4-Chlorophenyl), *N*-(3-methylphenyl)-succinamide monohydrate was filtered under suction, washed thoroughly with water, dilute sodium hydroxide solution and finally with water. It was recrystallized to constant melting point from a mixture of acetone and chloroform. The purity of the compound was checked and characterized by its infrared and NMR spectra.

Prism like colorless single crystals used in the X-ray diffraction studies were grown in 1:1 mixture of acetone and chloroform at room temperature.

### Refinement

The H atoms of the NH groups and the H atoms of the water molecule were located in a difference map and later restrained to the distance N—H = 0.86 (2) Å and O—H = 0.85 (2) Å, respectively. The other H atoms were positioned with idealized geometry using a riding model with the aromatic C—H = 0.93 Å, the methyl C—H = 0.96Å and the methylene C—H = 0.97 Å.

All H atoms were refined with isotropic displacement parameters. The *U*_iso_(H) values were set at 1.2*U*_eq_(C-aromatic, N) and 1.5*U*_eq_(*C*-methyl).

### Crystal data

**Table:**

C_17_H_17_ClN_2_O_2_·H_2_O	*F*(000) = 704
*M**_r_* = 334.79	*D*_x_ = 1.372 Mg m^−^^3^
Monoclinic, *P*2_1_/*n*	Mo *K*α radiation, λ = 0.71073 Å
Hall symbol: -P 2yn	Cell parameters from 1575 reflections
*a* = 12.210 (1) Å	θ = 2.9–27.9°
*b* = 4.9111 (5) Å	µ = 0.25 mm^−^^1^
*c* = 27.078 (3) Å	*T* = 293 K
β = 93.104 (9)°	Prism, colourless
*V* = 1621.3 (3) Å^3^	0.36 × 0.28 × 0.08 mm
*Z* = 4	

### Data collection

**Table:**

Oxford Diffraction Xcalibur Single Crystal X-ray Diffractometer with Sapphire CCD Detector	3297 independent reflections
Radiation source: fine-focus sealed tube	2047 reflections with *I* > 2σ(*I*)
graphite	*R*_int_ = 0.027
Rotation method data acquisition using ω scans.	θ_max_ = 26.4°, θ_min_ = 2.9°
Absorption correction: multi-scan (*CrysAlis RED*; Oxford Diffraction, 2009)	*h* = −15→12
*T*_min_ = 0.915, *T*_max_ = 0.980	*k* = −6→4
5592 measured reflections	*l* = −33→28

### Refinement

**Table:**

Refinement on *F*^2^	Primary atom site location: structure-invariant direct methods
Least-squares matrix: full	Secondary atom site location: difference Fourier map
*R*[*F*^2^ > 2σ(*F*^2^)] = 0.053	Hydrogen site location: inferred from neighbouring sites
*wR*(*F*^2^) = 0.127	H atoms treated by a mixture of independent and constrained refinement
*S* = 1.02	*w* = 1/[σ^2^(*F*_o_^2^) + (0.0476*P*)^2^ + 0.9732*P*] where *P* = (*F*_o_^2^ + 2*F*_c_^2^)/3
3297 reflections	(Δ/σ)_max_ = 0.006
221 parameters	Δρ_max_ = 0.37 e Å^−^^3^
2 restraints	Δρ_min_ = −0.21 e Å^−^^3^

### Special details

**Table:**

Geometry. All e.s.d.'s (except the e.s.d. in the dihedral angle between two l.s. planes) are estimated using the full covariance matrix. The cell e.s.d.'s are taken into account individually in the estimation of e.s.d.'s in distances, angles and torsion angles; correlations between e.s.d.'s in cell parameters are only used when they are defined by crystal symmetry. An approximate (isotropic) treatment of cell e.s.d.'s is used for estimating e.s.d.'s involving l.s. planes.
Refinement. Refinement of *F*^2^ against ALL reflections. The weighted *R*-factor *wR* and goodness of fit *S* are based on *F*^2^, conventional *R*-factors *R* are based on *F*, with *F* set to zero for negative *F*^2^. The threshold expression of *F*^2^ > σ(*F*^2^) is used only for calculating *R*-factors(gt) *etc*. and is not relevant to the choice of reflections for refinement. *R*-factors based on *F*^2^ are statistically about twice as large as those based on *F*, and *R*- factors based on ALL data will be even larger.

### Fractional atomic coordinates and isotropic or equivalent isotropic displacement parameters (Å^2^)

**Table:**

	*x*	*y*	*z*	*U*_iso_*/*U*_eq_	
Cl1	0.34943 (7)	−0.42307 (17)	0.47553 (3)	0.0622 (3)	
O1	0.62296 (14)	0.4094 (4)	0.31577 (7)	0.0451 (5)	
O2	0.47762 (15)	1.1220 (4)	0.19755 (8)	0.0554 (6)	
N1	0.43883 (17)	0.4107 (5)	0.32510 (8)	0.0380 (5)	
H1N	0.3799 (17)	0.487 (5)	0.3143 (10)	0.046*	
N2	0.65157 (17)	1.1254 (5)	0.17399 (8)	0.0373 (6)	
H2N	0.7152 (16)	1.057 (5)	0.1796 (10)	0.045*	
C1	0.4245 (2)	0.2068 (5)	0.36099 (9)	0.0351 (6)	
C2	0.3184 (2)	0.1093 (6)	0.36530 (11)	0.0457 (7)	
H2	0.2618	0.1769	0.3444	0.055*	
C3	0.2956 (2)	−0.0856 (6)	0.39993 (11)	0.0480 (8)	
H3	0.2246	−0.1509	0.4021	0.058*	
C4	0.3790 (2)	−0.1817 (6)	0.43102 (10)	0.0410 (7)	
C5	0.4841 (2)	−0.0908 (6)	0.42698 (11)	0.0510 (8)	
H5	0.5402	−0.1603	0.4479	0.061*	
C6	0.5079 (2)	0.1033 (6)	0.39211 (11)	0.0469 (7)	
H6	0.5796	0.1638	0.3896	0.056*	
C7	0.5316 (2)	0.4993 (5)	0.30498 (9)	0.0322 (6)	
C8	0.5080 (2)	0.7174 (6)	0.26629 (10)	0.0398 (7)	
H8A	0.4700	0.8660	0.2816	0.048*	
H8B	0.4585	0.6421	0.2406	0.048*	
C9	0.6068 (2)	0.8327 (6)	0.24219 (10)	0.0379 (6)	
H9A	0.6470	0.6861	0.2274	0.046*	
H9B	0.6552	0.9190	0.2671	0.046*	
C10	0.5724 (2)	1.0393 (5)	0.20277 (10)	0.0356 (6)	
C11	0.6394 (2)	1.2938 (5)	0.13128 (9)	0.0336 (6)	
C12	0.5546 (2)	1.4811 (5)	0.12350 (10)	0.0371 (6)	
H12	0.5030	1.5018	0.1473	0.044*	
C13	0.5460 (2)	1.6381 (5)	0.08064 (10)	0.0400 (7)	
C14	0.6241 (2)	1.6058 (6)	0.04607 (11)	0.0515 (8)	
H14	0.6194	1.7083	0.0172	0.062*	
C15	0.7089 (3)	1.4234 (7)	0.05399 (11)	0.0536 (8)	
H15	0.7611	1.4055	0.0304	0.064*	
C16	0.7176 (2)	1.2668 (6)	0.09629 (10)	0.0436 (7)	
H16	0.7752	1.1444	0.1013	0.052*	
C17	0.4529 (3)	1.8381 (6)	0.07173 (12)	0.0548 (8)	
H17A	0.4172	1.8673	0.1020	0.066*	
H17B	0.4011	1.7662	0.0472	0.066*	
H17C	0.4814	2.0078	0.0603	0.066*	
O3	0.26720 (16)	0.1047 (4)	0.22674 (8)	0.0468 (5)	
H31	0.334 (3)	0.112 (6)	0.2162 (11)	0.056*	
H32	0.255 (3)	0.256 (7)	0.2390 (12)	0.056*	

### Atomic displacement parameters (Å^2^)

**Table:**

	*U*^11^	*U*^22^	*U*^33^	*U*^12^	*U*^13^	*U*^23^
Cl1	0.0730 (6)	0.0536 (5)	0.0616 (5)	−0.0016 (4)	0.0197 (4)	0.0174 (4)
O1	0.0285 (10)	0.0509 (12)	0.0559 (12)	−0.0002 (9)	0.0033 (8)	0.0104 (10)
O2	0.0303 (10)	0.0670 (15)	0.0701 (14)	0.0120 (10)	0.0148 (9)	0.0289 (12)
N1	0.0273 (11)	0.0412 (13)	0.0457 (13)	0.0033 (11)	0.0043 (10)	0.0110 (11)
N2	0.0237 (11)	0.0434 (14)	0.0452 (13)	0.0038 (10)	0.0050 (10)	0.0084 (11)
C1	0.0330 (14)	0.0361 (15)	0.0368 (14)	−0.0021 (12)	0.0066 (11)	0.0001 (12)
C2	0.0331 (14)	0.0499 (18)	0.0541 (18)	−0.0007 (14)	0.0023 (13)	0.0109 (15)
C3	0.0371 (15)	0.0505 (19)	0.0575 (18)	−0.0076 (14)	0.0113 (14)	0.0078 (16)
C4	0.0469 (17)	0.0359 (15)	0.0415 (16)	−0.0018 (13)	0.0133 (13)	0.0021 (13)
C5	0.0460 (17)	0.0529 (19)	0.0538 (18)	0.0040 (15)	0.0008 (14)	0.0168 (16)
C6	0.0322 (14)	0.0548 (19)	0.0536 (17)	−0.0029 (14)	0.0000 (13)	0.0136 (15)
C7	0.0278 (13)	0.0325 (14)	0.0364 (14)	−0.0034 (11)	0.0025 (11)	−0.0036 (11)
C8	0.0350 (14)	0.0384 (15)	0.0465 (16)	0.0006 (13)	0.0072 (12)	0.0058 (13)
C9	0.0300 (13)	0.0387 (15)	0.0452 (16)	−0.0001 (12)	0.0032 (12)	0.0049 (13)
C10	0.0282 (13)	0.0363 (15)	0.0426 (15)	0.0008 (12)	0.0057 (11)	0.0002 (12)
C11	0.0305 (13)	0.0323 (14)	0.0380 (14)	−0.0048 (12)	0.0012 (11)	0.0014 (12)
C12	0.0329 (14)	0.0377 (15)	0.0410 (15)	−0.0004 (12)	0.0058 (12)	−0.0033 (12)
C13	0.0401 (15)	0.0326 (15)	0.0465 (16)	−0.0006 (12)	−0.0044 (12)	0.0007 (13)
C14	0.0543 (18)	0.0526 (19)	0.0479 (17)	0.0000 (16)	0.0051 (15)	0.0115 (15)
C15	0.0505 (18)	0.064 (2)	0.0482 (18)	0.0066 (17)	0.0172 (14)	0.0094 (17)
C16	0.0334 (14)	0.0494 (18)	0.0487 (17)	0.0051 (13)	0.0077 (13)	0.0059 (14)
C17	0.0543 (19)	0.0449 (18)	0.064 (2)	0.0063 (16)	−0.0041 (16)	0.0057 (16)
O3	0.0288 (10)	0.0451 (12)	0.0670 (14)	−0.0026 (10)	0.0065 (9)	−0.0049 (11)

### Geometric parameters (Å, °)

**Table:**

Cl1—C4	1.742 (3)	C8—H8A	0.9700
O1—C7	1.220 (3)	C8—H8B	0.9700
O2—C10	1.227 (3)	C9—C10	1.516 (4)
N1—C7	1.355 (3)	C9—H9A	0.9700
N1—C1	1.413 (3)	C9—H9B	0.9700
N1—H1N	0.849 (17)	C11—C16	1.388 (3)
N2—C10	1.343 (3)	C11—C12	1.392 (4)
N2—C11	1.423 (3)	C12—C13	1.392 (4)
N2—H2N	0.852 (17)	C12—H12	0.9300
C1—C6	1.382 (4)	C13—C14	1.381 (4)
C1—C2	1.393 (3)	C13—C17	1.512 (4)
C2—C3	1.379 (4)	C14—C15	1.377 (4)
C2—H2	0.9300	C14—H14	0.9300
C3—C4	1.369 (4)	C15—C16	1.379 (4)
C3—H3	0.9300	C15—H15	0.9300
C4—C5	1.369 (4)	C16—H16	0.9300
C5—C6	1.384 (4)	C17—H17A	0.9600
C5—H5	0.9300	C17—H17B	0.9600
C6—H6	0.9300	C17—H17C	0.9600
C7—C8	1.515 (4)	O3—H31	0.88 (3)
C8—C9	1.513 (3)	O3—H32	0.83 (3)
			
C7—N1—C1	129.9 (2)	C8—C9—H9A	109.5
C7—N1—H1N	115.5 (19)	C10—C9—H9A	109.5
C1—N1—H1N	114.6 (19)	C8—C9—H9B	109.5
C10—N2—C11	127.4 (2)	C10—C9—H9B	109.5
C10—N2—H2N	116.8 (19)	H9A—C9—H9B	108.0
C11—N2—H2N	115.4 (19)	O2—C10—N2	122.2 (3)
C6—C1—C2	118.7 (2)	O2—C10—C9	121.8 (2)
C6—C1—N1	124.4 (2)	N2—C10—C9	116.0 (2)
C2—C1—N1	116.8 (2)	C16—C11—C12	119.6 (2)
C3—C2—C1	121.2 (3)	C16—C11—N2	117.0 (2)
C3—C2—H2	119.4	C12—C11—N2	123.5 (2)
C1—C2—H2	119.4	C11—C12—C13	120.9 (2)
C4—C3—C2	119.1 (3)	C11—C12—H12	119.5
C4—C3—H3	120.4	C13—C12—H12	119.5
C2—C3—H3	120.4	C14—C13—C12	118.5 (3)
C3—C4—C5	120.5 (3)	C14—C13—C17	120.5 (3)
C3—C4—Cl1	119.0 (2)	C12—C13—C17	121.0 (2)
C5—C4—Cl1	120.5 (2)	C15—C14—C13	120.6 (3)
C4—C5—C6	120.8 (3)	C15—C14—H14	119.7
C4—C5—H5	119.6	C13—C14—H14	119.7
C6—C5—H5	119.6	C14—C15—C16	121.0 (3)
C1—C6—C5	119.6 (3)	C14—C15—H15	119.5
C1—C6—H6	120.2	C16—C15—H15	119.5
C5—C6—H6	120.2	C15—C16—C11	119.3 (3)
O1—C7—N1	124.1 (2)	C15—C16—H16	120.4
O1—C7—C8	123.9 (2)	C11—C16—H16	120.4
N1—C7—C8	111.9 (2)	C13—C17—H17A	109.5
C7—C8—C9	115.9 (2)	C13—C17—H17B	109.5
C7—C8—H8A	108.3	H17A—C17—H17B	109.5
C9—C8—H8A	108.3	C13—C17—H17C	109.5
C7—C8—H8B	108.3	H17A—C17—H17C	109.5
C9—C8—H8B	108.3	H17B—C17—H17C	109.5
H8A—C8—H8B	107.4	H31—O3—H32	106 (3)
C8—C9—C10	110.9 (2)		
			
C7—N1—C1—C6	17.5 (5)	C11—N2—C10—O2	−7.3 (5)
C7—N1—C1—C2	−163.6 (3)	C11—N2—C10—C9	172.6 (2)
C6—C1—C2—C3	0.3 (4)	C8—C9—C10—O2	9.3 (4)
N1—C1—C2—C3	−178.6 (3)	C8—C9—C10—N2	−170.6 (2)
C1—C2—C3—C4	0.9 (5)	C10—N2—C11—C16	−153.7 (3)
C2—C3—C4—C5	−1.7 (5)	C10—N2—C11—C12	26.7 (4)
C2—C3—C4—Cl1	178.8 (2)	C16—C11—C12—C13	1.3 (4)
C3—C4—C5—C6	1.2 (5)	N2—C11—C12—C13	−179.1 (2)
Cl1—C4—C5—C6	−179.2 (2)	C11—C12—C13—C14	−0.6 (4)
C2—C1—C6—C5	−0.8 (4)	C11—C12—C13—C17	178.9 (3)
N1—C1—C6—C5	178.1 (3)	C12—C13—C14—C15	−0.3 (5)
C4—C5—C6—C1	0.0 (5)	C17—C13—C14—C15	−179.9 (3)
C1—N1—C7—O1	−0.1 (5)	C13—C14—C15—C16	0.6 (5)
C1—N1—C7—C8	178.2 (3)	C14—C15—C16—C11	0.1 (5)
O1—C7—C8—C9	−2.7 (4)	C12—C11—C16—C15	−1.0 (4)
N1—C7—C8—C9	179.1 (2)	N2—C11—C16—C15	179.4 (3)
C7—C8—C9—C10	177.5 (2)		

### Hydrogen-bond geometry (Å, °)

**Table:**

*D*—H···*A*	*D*—H	H···*A*	*D*···*A*	*D*—H···*A*
N1—H1N···O3^i^	0.85 (2)	2.14 (2)	2.971 (3)	166 (3)
N2—H2N···O1^ii^	0.85 (2)	2.10 (2)	2.949 (3)	172 (3)
O3—H31···O2^iii^	0.88 (3)	1.86 (3)	2.730 (3)	177 (3)
O3—H32···O3^i^	0.83 (3)	1.97 (3)	2.802 (2)	175 (3)

Symmetry codes: (i) −*x*+1/2, *y*+1/2, −*z*+1/2; (ii) −*x*+3/2, *y*+1/2, −*z*+1/2; (iii) *x*, *y*−1, *z*.
